# Spray-Dried Microcapsules of Lavandin (*Lavandula* × *intermedia*) Essential Oil: Formulation and Functional Properties

**DOI:** 10.3390/molecules30204098

**Published:** 2025-10-15

**Authors:** Jelena Bajac, Milena Terzić, Branislava Nikolovski, Lidija Petrović, Branimir Bajac, Gökhan Zengin, Ivana Mitrović

**Affiliations:** 1 Faculty of Technology Novi Sad, University of Novi Sad, Bulevar cara Lazara 1, 21000 Novi Sad, Serbia; ilicj@uns.ac.rs (J.B.); barjakb@uns.ac.rs (B.N.); lidijap@uns.ac.rs (L.P.); tadi@uns.ac.rs (I.M.); 2 BioSense Institute, University of Novi Sad, Zorana Djindjića 1, 21000 Novi Sad, Serbia; branimir.bajac@biosense.rs; 3 Science Faculty, Selcuk University, Campus Konya, 42130 Konya, Turkey; biyologzengin@gmail.com

**Keywords:** Lavandin essential oil, *Lavandula* × *intermedia*, spray drying microencapsulation, encapsulation efficiency, antimicrobial activity, antioxidant potential, enzyme inhibition

## Abstract

Lavandin essential oil (LEO) (*Lavandula* × *intermedia*) is a high-yielding aromatic product with broad bioactive potential, but its direct application is hindered by its volatility, rapid oxidation, and environmental sensitivity. In this study, the microencapsulation of LEO by spray drying using different wall materials was investigated: Maltodextrin (MD), Gum Arabic (GA), Whey Protein Concentrate (WPC), Inulin (IN), and Modified Starch (Hi-Cap). The resulting formulations exhibited encapsulation efficiencies (EEs) of 55.35–83.29%, oil retention (RE) of 49.07–76.65%, and yields of 41.39–71.47%. The MD/GA blend with Tween 80 performed best, as it offered high EE and RE, low residual moisture, fast reconstitution, and strong protection of the encapsulated oil against thermal and moisture stress. Gas chromatography–mass spectrometry (GC–MS) identified 38 volatile components, with linalyl acetate (30.38%) and linalool (24.65%) being the major components. Biological tests confirmed that the antimicrobial and antifungal activity of lavandin against some pathogens was maintained even when a much lower concentration of the oil (1–5%) was used in encapsulated form. Antioxidant activity decreased after encapsulation, while tyrosinase inhibition increased, indicating cosmetic potential. These results show that spray drying is an effective strategy for stabilizing LEO and expanding its applications in various industries.

## 1. Introduction

Essential oils (EOs) represent a high-value segment of the natural products market, as they are attractive for applications in food preservation, pharmaceuticals, cosmetics, and agriculture due to their extensive bioactivity, antimicrobial, antioxidant, anti-anxiety, anti-inflammatory, and insecticidal properties [1]. The global essential oils market is estimated to have reached US$12.47 billion in 2024. Further forecasts indicate growth from US$13.66 billion in 2025 to US$27.82 billion in 2032, representing a compound annual growth rate (CAGR) of 10.69% during the forecast period. Europe was the leading regional market in 2024 with a 43.46% share of the total market [2]. Within this market, lavender and lavandin oils are among the most widely traded oils, valued for their distinctive scents and therapeutic properties.

Lavandin essential oil (LEO), extracted from *Lavandula* × *intermedia*, a cross between *L. angustifolia* and *L. latifolia*, is particularly valued in the fragrance and cleaning industry due to its high yield and robust fragrance profile. Compared to true lavender oil, lavandin EO is cheaper to produce and offers up to three times higher yield per hectare, making it economically attractive for large-scale industrial applications [3]. Its chemical composition, rich in linalool, linalyl acetate, camphor, and 1,8-cineole, lends itself to a broad spectrum of biological activity and improved stability in formulated products [4]. France and Bulgaria are leading producers of lavandin with an annual production of more than 1500 tonnes of essential oil, contributing significantly to the European aromatic plant economy [5].

However, despite its economic potential, the commercial utilization of LEO is hampered by its physicochemical instability. Like other essential oils, it is susceptible to oxidation, evaporation, photodegradation and chemical changes such as hydrolysis of linalyl acetate to linalool, resulting in loss of aroma, reduced shelf life and decreased bioactivity during storage and processing [6,7]. These issues are particularly critical in value-added applications, including natural preservatives, cosmetics and functional foods, where consistency and efficacy are critical. Microencapsulation has been shown to protect labile compounds and reduce such transformations, thereby preserving the chemical stability and bioactivity of the oil [8].

To overcome these challenges, microencapsulation has emerged as a leading strategy to improve EO stability, protect the active ingredients, and provide controlled release under the desired environmental conditions [9]. Among the different encapsulation techniques, spray drying is the most widely used industrially and accounts for over 80% of commercial encapsulation processes in the food and nutraceutical sector [10]. Spray drying offers several economic and technical advantages: low operating costs, scalability, fast processing, and the ability to influence particle size and release behavior by adjusting the formulation [11].

The encapsulation of LEO by spray drying not only improves its storage stability and ease of incorporation into powdered formulations but also increases its competitiveness in the market by reducing losses during transport and storage. In addition, spray-dried LEO products can fulfill the increasing consumer demand for sustainable, environmentally friendly, and plant-based functional ingredients that continues to grow in global markets [12]. More than 60% of European consumers are looking for natural flavors and therapeutic actives in personal care and food products, adding to the industry’s interest in stabilized essential oil formats [13].

However, the unique terpenoid content of lavandin and their high oxidative sensitivity require customized, tailored encapsulation conditions and formulation strategies. Key factors, such as the choice of wall materials, core-to-wall ratio, drying temperatures, and emulsion stability, have a significant impact on the encapsulation efficiency, morphology, and functional potential of the final powders [14].

Therefore, the main objective of this study was the encapsulation of lavandin (*Lavandula* × *intermedia*) essential oil by spray drying with a systematic evaluation of different wall materials (maltodextrin, gum arabic, whey protein concentrate, inulin, Hi-Cap) to determine the optimal emulsion matrix that allows efficient microencapsulation. A comprehensive physicochemical characterization of the resulting powders was performed, including oil retention efficiency (RE), encapsulation efficiency (EE), moisture content, hygroscopicity, dissolution time, flowability and cohesiveness, thermal stability, and surface morphology. In addition, the chemical composition and biological activities of both the native and microencapsulated LEO were analyzed. These studies included antioxidant capacity, inhibitory effects on key enzymes involved in metabolic and neurodegenerative diseases, and antimicrobial and antifungal activity against a range of selected pathogenic microorganisms. Overall, these efforts aim to provide a comprehensive overview of the development of multifunctional bioactive formulations that can be used in the pharmaceutical, cosmetic, and food industries.

## 2. Results and Discussion

### 2.1. Chemical Composition of Essential Oil

The chemical composition of LEO extracted from *Lavandula* × *intermedia* was comprehensively characterized by gas chromatography–mass spectrometry (GC-MS) in this study. A total of 38 volatile components were identified, comprising mainly oxygenated monoterpenes and monoterpene hydrocarbons (Table 1). The major components included linalyl acetate (30.38%), linalool (24.65%), camphor (8.80%), borneol (6.66%), terpinen-4-ol (4.58%), and 1,8-cineole (2.65%). The chemical composition of the essential oil analyzed in the present study shows strong agreement with previously reported data, in particular with the results of [3,15,16], who elucidated the phytochemical profiles of essential oils from *L. officinalis* and *L. angustifolia*. In all cases, linalyl acetate and linalool were identified as the predominant constituents, accompanied by a spectrum of other terpenoid compounds present in comparatively lower concentrations. In addition, the chemical composition determined was compared with published profiles of essential oils obtained from other *Lavandula* species from different geographical regions. In samples of *L. viridis* from Portugal, 1,8-cineole was identified as the main constituent [17]. In contrast, the essential oil of *L. dentata* collected in Algeria was characterized by high levels of β-pinene and myrtenol as dominant components [18]. In addition, the analysis of the essential oil of *L. intermedia* from Romania revealed camphor and eucalyptol as main constituents [19].

The two main constituents of most LEO (linalool and linalyl acetate) contribute to the aroma of EOs. They are often associated with the antioxidant, anti-inflammatory, analgesic, and anti-anxiety effects of lavender essential oils [20]. The high content of 1,8-cineole, camphor, and borneol is characteristic of hybrid lavandin essential oils, resulting in the hybrid species of LEO being less valued in perfumery but more effective as antimicrobials [21]. These interspecific and geographical variations emphasize the considerable phytochemical diversity within the genus *Lavandula*. The variability in the chemical composition of essential oils among plant species within the same genus primarily arises from genetic differences that govern biosynthetic pathways and determine species or chemotype-specific profiles [22].

Additionally, developmental stage and organ-specific expression significantly influence metabolite accumulation. Secondary factors such as environmental conditions (e.g., climate, soil, altitude), geographical origin, harvesting time, post-harvest handling, and extraction methodology also contribute to compositional differences. Furthermore, biotic stressors can induce the production of specific volatiles, adding to the phytochemical diversity observed across species. These combined factors underscore the complex interplay between genetic and environmental influences in shaping essential oil profiles [22].

**Table 1:** The main constituents identified in the essential oil of lavandin (LEO).

No	RI ^a^	RI ^b^	Compound	Content (%)
1	930	930	α-Thujene	0.38 ± 0.11
2	939	938	α-Pinene	1.67 ± 0.54
3	954	953	Camphene	0.63 ± 0.34
4	975	975	Sabinene	0.45 ± 0.15
5	979	980	β-Pinene	1.27 ± 0.53
6	979	980	1-Octen-3-ol	0.43 ± 0.15
7	990	993	β-Myrcene	1.83 ± 0.44
8	1011	1013	3-Carene	0.12 ± 0.08
9	1024	1025	p-Cymene	0.33 ± 0.12
10	1029	1031	Limonene	1.73 ± 0.66
11	1031	1034	Eucalyptol (1,8-Cineole)	2.65 ± 0.23
12	1037	1038	(Z)-β-Ocimene	1.35 ± 0.32
13	1059	1064	γ-Terpinene	0.44 ± 0.14
14	1072	1071	Linalool oxide	0.04 ± 0.08
15	1088	1088	Terpinolene	0.19 ± 0.23
16	1096	1099	Linalool	24.65 ± 2.51
17	1116	1115	Fenchol	0.12 ± 0.06
18	1146	1145	Camphor	8.80 ± 2.83
19	1169	1167	Borneol	6.66 ± 2.62
20	1169	1165	Lavandulol	0.51 ± 0.78
21	1177	1177	Terpinen-4-ol	4.58 ± 1.63
22	1188	1189	α-Terpineol	1.10 ± 0.45
23	1192	1193	Hexyl butanoate	0.54 ± 0.17
24	1195	1197	Myrtenal	0.01 ± 0.02
25	1237	1233	Pulegone	0.05 ± 0.07
26	1243	1243	Carvone	0.20 ± 0.05
27	1257	1256	Linalyl acetate	30.38 ± 9.53
28	1271	1289	Lavandulol acetate	2.76 ± 0.94
29	1361	1365	Neryl acetate	0.40 ± 0.09
30	1376	1376	α-copaene	0.68 ± 0.36
31	1388	1385	β-Bourbonene	0.07 ± 0.07
32	1417	1427	Santalene	0.13 ± 0.01
33	1419	1420	Caryophyllene	1.25 ± 1.20
34	1442	1442	(Z)-β-Farnesene	1.22 ± 0.73
35	1583	1583	Caryophyllene oxide	0.50 ± 0.36
36	1640	1640	τ-Cadinol	0.01 ± 0.02
37	1685	1685	α-Bisabolol	0.18 ± 0.13

^a^ An experiment retention indices (HP-5 MS column). ^b^ the most common literature retention indices (HP-5MS column) according to NIST—library database—[23].

Concerning the stabilization, formulation, and development of new products based on the essential oil of *Lavandula* × *intermedia*, the pronounced volatility and chemical instability of its terpene components pose a major technological challenge. The most important compounds, such as linalyl acetate, linalool, camphor, and borneol, have well-documented biological activities, including anti-inflammatory, antioxidant, antimicrobial, and anticarcinogenic effects [24]. Therefore, the preservation of these constituents is crucial for maintaining the overall quality and therapeutic potential of the essential oil. However, their high susceptibility to degradation by environmental factors such as light, oxygen, and high temperatures can lead to a significant loss of bioactivity. To mitigate these problems and facilitate the development of stable and functional formulations, microencapsulation has proven to be a highly effective strategy. This approach not only protects the essential oil from physicochemical degradation but also enables controlled release and improved integration into various delivery systems, ensuring both chemical integrity and biological efficacy [8,20].

### 2.2. Characterization of Lavandin Essential Oil Microcapsules

The results of the characterization of the LEO microcapsules, which include the determination of the total oil content, the surface oil content, the oil retention efficiency (RE), the encapsulation efficiency (EE), and the powder yield, are shown in Table 2. The moisture content, dissolution time, bulk density, tapped density, particle density, Carr’s index (CI), Hausner ratio (HR), and porosity of LEO microcapsules prepared with different carriers are shown in Table 3.

#### 2.2.1. Oil Retention Efficiency

The most important quality parameter for LEO microencapsulation is the oil retention efficiency, i.e., the percentage of the initial amount of essential oil trapped in the microcapsules [25]. The LEO retention values ranged from 49.07% to 76.65%. The main factor for oil retention is the selection of wall material. Among the carriers analyzed, GA is the most commonly used encapsulation material for EOs, allowing high oil loading [25,26]. Its use is partly limited by its high price and the differences in GA structure due to its origin and manufacturing process [27,28]. Therefore, mixing GA with other carriers is a promising strategy for oil encapsulation. On the other hand, maltodextrin is an inexpensive encapsulation carrier but without emulsifying properties for the encapsulation of hydrophobic ingredients [27]. The stability of the feed emulsion is an essential prerequisite for efficient oil binding, i.e., a higher stability of the formed emulsion leads to a higher encapsulation efficiency and a lower oil loss [25]. Therefore, the preparation of an LEO1 feed emulsion using only MD as a carrier requires the addition of emulsifiers (Tween 80) to stabilize the droplets. When GA is used as a carrier, the presence of an emulsifier is not required, as GA itself has surface-active properties. Accordingly, the results presented in Table 2 show that there is no significant difference between the oil retention capacity of LEO powders produced with MD and MD/GA, with GA type having no significant effect on RE. The addition of Tween 80 to the MD/GA formulation resulted in a slight increase in RE, but this was still insignificant compared to LEO1 and LEO4. Replacing GA in the feed emulsion with WPC or IN (LEO5 and LEO6) resulted in a significant decrease in RE. On the other hand, the formulation with GA/IN (LEO7) showed a slightly higher RE, with similar results to the MD/GA formulation. The most effective carrier for the encapsulation of LEO was Hi-Cup, which contained the most considerable amount of entrapped lavandin oil (76.65 ± 0.25%).

#### 2.2.2. Encapsulation Efficiency

The encapsulation efficiency (EE), which refers to the amount of LEO successfully encapsulated in the microcapsules, ranged from 55.35 to 83.29%. Several previous studies have confirmed the strong influence of wall materials on the encapsulation efficiency of lavender essential oil [29,30]. The formulation with the highest surface oil content was LEO7, followed by LEO2 and LEO8. Surface oil content is an important parameter, as it indicates the amount of oil exposed to oxidation and external influences, which can affect the flavor of the powder [31]. Surprisingly, LEO1 had the lowest surface oil content, resulting in the highest encapsulation efficiency. The MD/GA microcapsules exhibited significantly lower EE than MD powder due to the higher oil content localized on the surface of the microcapsule. GA1 was favored over GA2 due to its statistically significant higher encapsulation efficiency. This is because the structure of GA directly affects its emulsifying properties and the viscosity of the feed emulsion, with additionally processed GA1 (in a mixture with MD) producing an emulsion with a significantly higher viscosity, resulting in a reduction in the time required for shell formation and oil movement within the droplets [27]. These physico-chemical differences between the GA types resulted in a higher total oil content and lower surface oil content. The addition of Tween 80 as an emulsifier in the MD/GA1 (LEO3) formulation significantly increased the encapsulation efficiency by influencing the increase in total oil content and the decrease in surface oil. There is competition between non-ionic surfactants and GA in the stabilization of emulsions, whereby Tween 80 can partially displace GA molecules from the oil-water interface [32]. This integration at the surface influences the decrease in interfacial tension, which likely has a direct impact on increasing encapsulation efficiency.

The use of WPC in the feed emulsion (LEO5) helps to reduce the surface oil content, resulting in a high EE of 77.37 ± 1.2%. The microcapsule LEO6 contained a small amount of total oil, but due to the low surface oil content, the EE was 72.34 ± 0.18%. However, it should be noted that 50% of the oil was lost during the spray-drying process. In contrast to these results, the LEO7 powder showed a higher total oil amount and a higher surface oil content, which led to a decrease in encapsulation efficiency. Hi-Cup as a carrier showed satisfactory results in microencapsulation of lavandin essential oil with an encapsulation efficiency of 70.54 ± 0.35%.

#### 2.2.3. Powder Yield

The encapsulation yield (Y) for the LEO microcapsules ranged from 41.39 to 71.47%. The formulation of the wall material significantly influenced the encapsulation yield, with the use of MD as a carrier (with Tween 80) resulting in the lowest loss of solid material. The yield of LEO1 differed significantly from the yields of all other formulations. Partial replacement of MD with other polymers in the emulsion formulations had an adverse effect on product yield, reducing it to 41.39%, 44.50%, 55.56%, and 65.51% for GA1, GA2, WPC, and IN, respectively. Previous studies have shown that maltodextrins in feed emulsions are often associated with higher yields due to their high solubility, which helps to reduce the hygroscopicity of the powder and prevent the material from settling and sticking in the spray drying chamber [30]. In the investigation [29] authors reported similar results in the encapsulation of lavender oil by spray drying using different ratios of MD and GA (42.10–63.50%).

#### 2.2.4. Moisture Content

The moisture content of the LEO microcapsules (Table 3) ranged from 1.12 to 4.92%. The formulation with MD and MD/IN as carriers had the lowest moisture content, with no significant differences between these two powder formulations. LEO microcapsules prepared with MD/GA showed significantly higher moisture content, which can be attributed to the influence of GA properties on droplet drying. GA as a material leads to rapid crust formation during the drying process and thus prevents the diffusion and evaporation of water during droplet drying [33,34]. The differences between the moisture values prepared with different GA types (LEO2 and LEO4) were significant, with the use of the additionally processed GA type (GA1) leading to an increase in moisture due to the higher water-holding capacity and higher viscosity of the feed emulsion [27]. However, the addition of Tween 80 in the formulation of the feed emulsion led to a significant reduction in the moisture content from 4.63 to 2.28%. Due to the altered structure of the interfacial layer caused by the addition of the surfactant, where some GA molecules were displaced by Tween 80 molecules [32], the influence of the fast-drying property of GA on particle moisture was probably lower. The formulation with MD/WPC (LEO 5) had a similar moisture content to LEO3. The powder prepared with GA1/IN had the highest moisture content, with no significant difference compared to the moisture content of the LEO4 powder.

#### 2.2.5. Dissolution Time

An important property of microcapsules for practical use is their good solubility in water. Powders with poor solubility can lead to difficulties in processing and economic losses [25]. The dissolution time of the LEO microcapsules varied from 0.82 to 6.93 min (Table 3), with LEO1 and LEO6 having the lowest values and differing significantly from all formulations analyzed. In contrast, the MD/WPC formulation (LEO5) had the highest dissolution time. This is consistent with the dissolution time of the juniper berry essential oil microcapsules from our earlier study [33].

#### 2.2.6. Bulk and Tapped Density

In powder production, the high bulk and tapped densities are desirable for transport and storage of the microencapsulated material, as smaller storage containers are required and lipid oxidation is prevented by smaller powder cavities for air ingress [35,36]. The LEO powders analyzed had bulk densities between 0.23 and 0.28 g/cm^3^, with the formulation with MD/IN (LEO 6) having the highest bulk density, with no significant differences compared to the LEO1 and LEO3 formulations. It is noticeable that all formulations contain Tween 80, which contributes to some extent to the increase in bulk density. The values obtained are similar to the bulk densities of microcapsules containing rosemary essential oil [37] and microcapsules containing pink pepper essential oil [36]. The tapped densities of the LEO microcapsules ranged from 0.4 to 0.53 g/cm^3^, with a higher value for the LEO1 formulation.

#### 2.2.7. Particle Density

The particle density of the microcapsules depends on the chemical composition and particle size of the powder and ranges from 0.85 to 1.83 g/cm^3^, for LEO microcapsules. The particle density of the powder prepared with MD (LEO1) and MD/WPC (LEO5) was significantly lower than that of all other formulations, indicating lighter particles. The highest value for particle density was found for the LEO4 formulation, indicating more compact particles. Similar values were obtained in our previous study during microencapsulation of juniper berry essential oil [33].

#### 2.2.8. Carr’s Index and Hausner Ratio

The mean values of Carr’s Index of the LEO powders were in the range of 38.86–51.81%, while the mean values of the Hausner ratio were between 1.63 and 2.08, which classifies them as powders with ‘awful’ flow properties [34]. The LEO microcapsules prepared with MD/IN (LEO6) have the smallest value of CI and HR but are still in the same category of flowability as all formulations studied. Previous studies also reported poor flowability of microcapsules in the microencapsulation of gurum seed oil [38], microencapsulation of fish oil [39], microencapsulation of refined kenaf seed oil [34], and microencapsulation of juniper berry essential oil [33] (CI values were 40.62–52.04, 37.0–51.5, 40.9–48.9, and 41.85–49.66%, respectively).

#### 2.2.9. Porosity

The porosity of the microcapsules is the property that indicates the storage stability of the powder. High porosity values mean a large space between the particles, which is a potential site for oxygen degradation processes [40]. In this study, the average porosity values of the LEO microcapsules ranged from 37.68 to 73.92% (Table 3). The formulation of the powder prepared with GA1 as a carrier had a significantly higher porosity value compared to the porosity of the other microcapsules. The addition of Tween 80 to the emulsion formulation with the MD/GA1 mixture decreased the porosity of the particles from 73.92% to 63.48%. Similar values were obtained in our previous study during the microencapsulation of juniper berry essential oil using the same carriers [33]. The use of WPC in the emulsion formulation also influenced the reduction in particle porosity. The microcapsules prepared using only MD as a carrier showed the lowest porosity of the LEO powder, with a significantly lower value compared to all other formulations.

#### 2.2.10. Hygroscopicity

**Figure 1:**
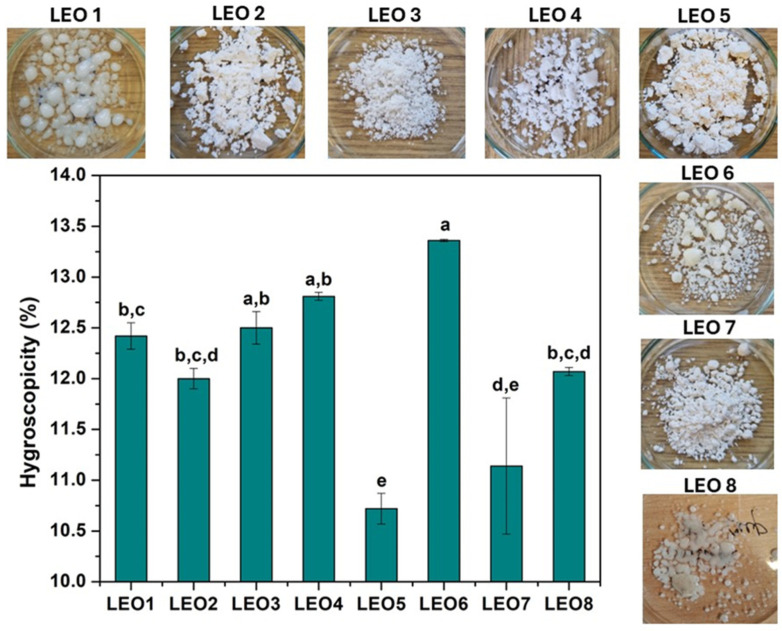
Hygroscopicity and appearance of LEO microcapsules prepared with different carrier formulations at 75.29% relative humidity and 25 °C. LEO1-MD; LEO2-MD/GA2 = 1:1 (*w*/*w*); LEO3-MD/GA1 = 1:1 (*w*/*w*) with 2% Tween80; LEO4-MD/GA1 = 1:1 (*w*/*w*); LEO5-MD/WPC = 1:1 (*w*/*w*); LEO6-MD/IN = 1:1 (*w*/*w*); LEO7-GA1/IN = 1:1 (*w*/*w*); LEO8-Hi-Cup. ^a,b,c,d,e^ Values with different letters differ significantly (*p* ≤ 0.05).

The hygroscopicity and appearance of the LEO microcapsules after exposure to 75% relative humidity are shown in Figure 1.

The hygroscopicity values were between 10.56 and 13.36% and can be categorized as slightly hygroscopic (10–15%) powders [41]. Too high a hygroscopicity value of the powder can lead to lipid oxidation and aggregation, which can alter the nutritional value and flow properties of the powder [34]. The hygroscopicity of LEO powders was lower than the hygroscopicity of rosemary essential oil microcapsules prepared with GA (15.87–18.90%) [37] and Cagaita fruit extract powders prepared with GA and IN [42] using the same drying process. The formulation with the lowest hygroscopicity values was LEO5, where the use of the WPC contributes to a decrease in moisture adsorption [33]. Previous studies have shown that the powders with the lowest moisture content consequently have the highest hygroscopicity, as the ability to absorb moisture is related to the water concentration gradient between the powder and the environment [34,43]. As can be seen, this study confirmed these observations, with the LEO6 formulation having a significantly lower moisture content, resulting in a high hygroscopicity of the sample. The hygroscopicity of the powder is directly related to the wall materials used, with the presence of GA and IN increasing water sorption. At the same time, the MD contributes to the reduction in hygroscopicity [44]. The present results confirm the influence of GA and IN on increasing the hygroscopicity of the powder, with the highest hygroscopicity occurring in the LEO6 formulation, with no significant difference between this formulation and LEO3 and LEO4. However, there is no apparent effect of MD on the reduction in hygroscopicity. The reason for this could be that an MD with a high DE was used for this study, whereby it is claimed that maltodextrins with a low dextrose equivalent are less hygroscopic than maltodextrins with a higher DE. They have a lower tendency to bind water [30]. In addition to the hygroscopicity values of the LEO powders, some samples exhibited strong swelling and melting at the analyzed relative humidity, resulting in a physical change to the obtained powders. As can be seen from Figure 1, the powders prepared with MD (LEO1) and Hi-Cup (LEO8) were melted entirely, while the formulation with MD/IN (LEO6) showed significant aggregation and swelling. The powders that showed no structural changes at a relative humidity of 75.29% were LEO3, LEO5, and LEO7, indicating which carriers can be characterized as wall materials suitable for the mass production of powders. For this reason, powders with pronounced changes in physical appearance are not considered further in this article.

### 2.3. Thermal Analysis of Lavandin Essential Oil Microcapsules

**Figure 2:**
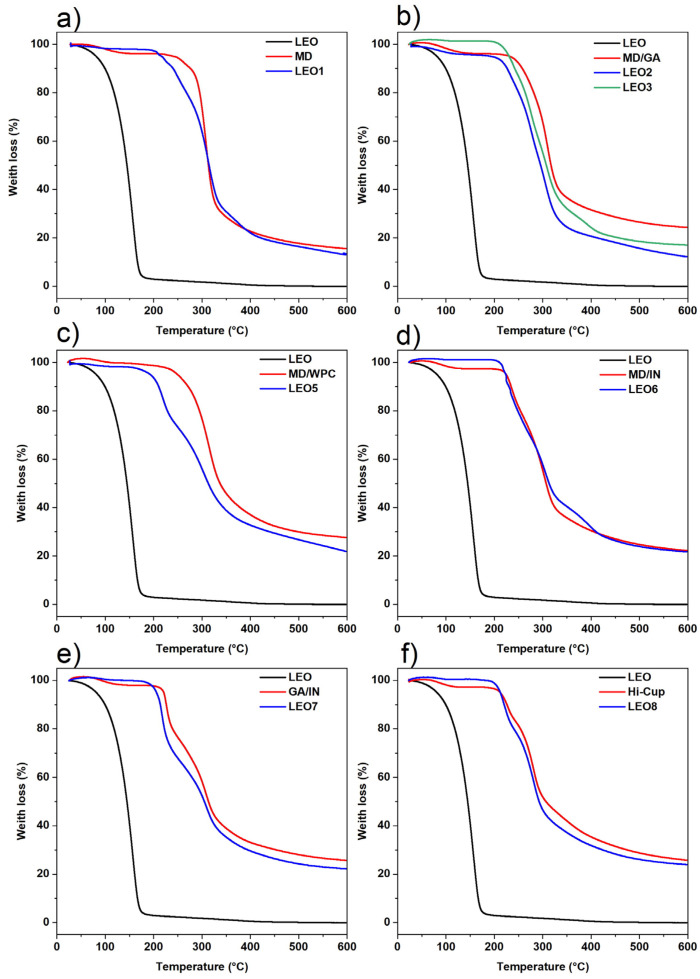
TGA curves of LEO, the used carriers and the microcapsules containing lavandin essential oil: (**a**) MD-Maltodextrin; (**b**) MD/GA-Maltodextrin/Gum arabic = 1:1 (*w*/*w*); (**c**) MD/WPC-maltodextrin/Whey protein concentrate = 1:1 (*w*/*w*); (**d**) MD/IN-Maltodextrin/Inulin = 1:1 (*w*/*w*); (**e**) GA/IN-Gum Arabic/Inulin = 1:1 (*w*/*w*); (**f**) Hi-Cup-Modified starch.

A TG analysis provided information on the thermal stability of the microcapsules (Figure 2). Each graph compares the TG curves of the LEO, the wall material, and the LEO microcapsules produced with the corresponding wall material. The LEO showed a slight decrease (<1%) in mass fraction at temperatures < 50 °C, a substantial loss (>10%) when the temperature exceeded 100 °C, and it decomposes completely below 200 °C. The low thermal stability of LEO indicates the great importance of encapsulation to preserve its biological properties [33,45,46].

All investigated carriers used for LEO microencapsulation can be used to protect the essential oil from thermal degradation, which is very important for the application of the microcapsules in further powder processing. No significant weight loss was observed when the microcapsules were thermally treated in the range where LEO vaporizes, i.e., between 100 and 200 °C. When using MD/GA as a wall material (Figure 2b), a slight weight loss of a few percent is observed around 100 °C, probably due to the presence of moisture [47] in the powder of the LEO2 formulation. The differences in the first part of these curves presented for LEO2 and LEO3 are due to the different moisture contents, caused by the differences in the moisture-binding capabilities of GA1 and GA2 [27]. From this point onwards, the shape and slope of the curve are identical regardless of the type of GA used. The thermal curve for the LEO4 formulation has not been shown, as it completely overlaps with LEO3, except in the initial part, where it overlaps with LEO2.

The microcapsules begin to lose weight slightly before the wall material decomposes. This fact corresponds to the evaporation of the encapsulated oil in the microcapsule and occurs immediately before the wall material begins to decompose. The temperatures at which encapsulated LEO start to evaporate are around 170, 205, and 215 °C for LEO5, LEO1, and LEO3 (LEO6), respectively. Therefore, the LEO3 and LEO6 formulations have a slight advantage in terms of thermal stability, but all other formulations can encapsulate and thermally protect LEO very effectively at temperatures below 200 °C.

### 2.4. Particle Size and Morphology of the LEO Microcapsules

The results of the LEO microcapsule characterization are used to select the optimal wall material for further investigation into the antimicrobial and antioxidant activities of the LEO powder. Based on this section, all further analyses were carried out exclusively with the LEO3 formulation. This decision was based on its overall superior performance among the carriers tested: LEO3 showed high encapsulation efficiency (78.77%), good oil retention capacity, optimal powder properties (moisture, dissolution time, density parameters) and, most importantly, greater stability under storage conditions compared to other formulations. In contrast, several powders (e.g., LEO1 and LEO8) showed pronounced instability at high relative humidity, while others (e.g., LEO2, LEO4, LEO7) were characterized by less favorable encapsulation yield or higher surface oil content, which could affect long-term stability. Therefore, LEO3 was selected as a representative sample for advanced analyses (particle size and morphology, antimicrobial activity, antioxidant potential and enzyme inhibition) as it offered the best balance between functionality and stability.

**Figure 3:**
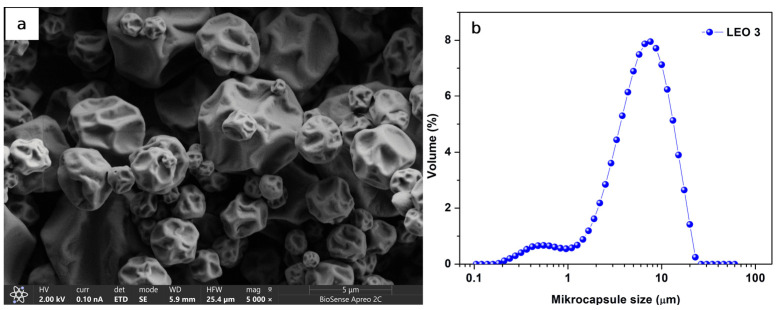
(**a**) SEM micrographs of the spray-dried LEO3 microcapsules and (**b**) volume particle size distribution of LEO3.

The mean particle size and morphology of the LEO microcapsules prepared with MD/GA, which were additionally stabilized with 2% Tween 80 (LEO3), are shown in Figure 3. As can be seen from Figure 3b, the particle size distribution curve is bimodal, with a small fraction of small particles (below 1 μm) and a significant fraction of particles below 10 μm. The mean particle size of the LEO3 powder was 7.52 ± 0.07 μm. A similar particle size of lavender essential oil was obtained by [28], who prepared microcapsules with a particle size in the range of 7–17 μm by spray drying at 150 °C and using MD/gum acacia as a carrier. In the [30] published paper, with slightly smaller mean particle sizes of lavender microcapsules (3.84 ± 0.21 µm for MD powder and 5.99 ± 0.22 µm for MD/GA powder additionally stabilized with 5% emulsifier) prepared by spray drying at an inlet temperature of 170 °C.

The morphological characteristics of the LEO3 microcapsules are shown in Figure 3a. As can be seen, the obtained LEO3 microparticles were semi-spherical and wrinkled, with dents but no cracks on the particle surface. The high inlet temperature and rapid evaporation through the wall material during particle formation may cause the irregular surface of the microcapsules [48]. The wrinkled surface of the particles can be helpful for some powder applications, as it allows better dispersibility and rehydration of the particles [49]. On the other hand, the presence of fractures, cracks, and defects on the surface of the microcapsules is not desirable, as they can lead to oxidation and degradation of the encapsulated essential oil [50].

### 2.5. Antimicrobial Potential of Lavandin Essential Oil Microcapsules

The antimicrobial efficacy of free and microencapsulated lavender essential oil (LEO) against Gram-positive (*B.cereus*, *E. faecalis*, *S. aureus*) and Gram-negative (*S. enterica*, *E. coli*, *K. pneumoniae*) strains is summarized in Table 4. Generally, free LEO outperformed microencapsulated preparations in agar diffusion, while microencapsulated LEO showed graded, concentration-dependent effects (1–5%). Considering that free essential oil is limited in practical use due to its organoleptic properties and hydrophobicity, encapsulated forms can be useful in preparations where prolonged release and protection from oxidation are important [51,52].

Gram-positive bacteria generally showed inhibition at lower microencapsulated concentrations. *B. cereus* responded from 3% upward but more strongly to free LEO. *E. faecalis* was inhibited already at 1% but showed a modest reduction with free LEO. This pattern can occur from assay variability, differential diffusion, or stress-response activation at very high terpenoid exposure. The literature commonly reports that Gram-positive bacteria are more sensitive to essential oils than Gram-negative bacteria, due to the absence of an outer membrane and a thick, porous peptidoglycan layer that does not impede hydrophobic terpenoids [53,54,55].

Gram-negative bacteria, with their lipopolysaccharide outer membrane, typically showed higher concentration thresholds. *S. enterica* exhibited no activity at ≤3% but apparent inhibition at 4–5% of microencapsulated LEO3 and a marked increase with free LEO. *K. pneumoniae* was largely unresponsive at low doses but showed moderate zones at higher levels and with free LEO (20.75 mm). Notably, *E. coli* followed the same threshold behavior at low doses yet displayed an enormous zone with free LEO (43.5 mm), underscoring that once the outer-membrane barrier is overcome either by high local concentrations, synergistic oil constituents, or improved diffusion, the intrinsic susceptibility of Gram-negative membranes to terpenoid-induced disorder becomes evident. This Gram-negative bacteria pattern is consistent with prior work showing that higher doses or permeabilization are often required to traverse the lipopolysaccharide barrier, after which essential oils rapidly disrupt inner membranes [52,53,55]. So, once terpenoids access the cytoplasmic membrane, both Gram-positive and Gram-negative are vulnerable to membrane disordering. However, Gram-positive walls lack the additional outer barrier, explaining their earlier response at lower microencapsulated percentages [52,54].

Also, the antifungal efficacy of free and microencapsulated LEO against most toxigenic phitopathogenic fungi (*F. graminearum*, *A. flavus*, *Penicillium* sp.) is summarized in Table 4. Among filamentous fungi, free LEO enabled huge zones against *F. graminearum* and *Penicillium* sp. These patterns align with known antifungal effects of lavender (rich in linalool and linalyl acetate), which disrupt ergosterol-containing membranes, increase permeability, and inhibit spore germination and hyphal extension [52,56]. At low microencapsulated concentrations, antifungal action was minimal, likely because the controlled release system restricted the instantaneous diffusion of volatile constituents through the agar matrix. This slow release may be advantageous in in vivo or food preservation contexts. Free oil, by contrast, allowed for rapid volatilization and diffusion, creating higher vapor-phase concentrations that are particularly effective against fungi with high aerial growth rates such as *Fusarium*.

Microencapsulated LEO3 demonstrated apparent antifungal effects, with potency increasing proportionally to concentration and reaching a maximum with free oil. The highest susceptibility was recorded for *F. graminearum* (40.75 mm) and *Penicillium* sp. (38.75 mm) with free LEO, while *A. flavus* exhibited moderate but still significant inhibition (21.5 mm). At lower microencapsulated doses (1–3%), inhibition was negligible or absent for most fungi, indicating that the release rate and local concentration of active volatiles are critical for antifungal efficacy.

The potent inhibition of *F. graminearum* and *Penicillium* suggests that LEO is primarily active against molds with permeable membranes. Previous studies have shown that *P. expansum* growth and mycotoxin production can be significantly reduced by lavender EO vapors, supporting the role of volatiles in post-harvest biocontrol [57].

Therefore, microencapsulation of essential oils enhances their utility in food and cosmetic protection by stabilizing volatile actives, shielding them from oxidation and light, and enabling controlled, sustained release. This fact improves antimicrobial efficacy over time while minimizing negative sensory impact (flavors/odors) and dermal irritation risk compared with free oils. Encapsulation also allows accurate dosing, better dispersibility in aqueous systems, and compatibility with packaging or coatings (e.g., edible films), forming localized antimicrobial barriers. These advantages enable longer shelf-life, making microencapsulated essential oils an attractive, multifunctional barrier in food safety and cosmetic preservation programs.

### 2.6. Antioxidant Potential

The antioxidant potential of LEO essential oil and microcapsules was evaluated using a comprehensive series of in vitro assays, including 2,2-diphenyl-1-picrylhydrazyl (DPPH), 2,2′-azino-bis-(3-ethylbenzothiazoline-6-sulfonic acid) (ABTS), copper ion reducing antioxidant capacity (CUPRAC), ferric reducing antioxidant power (FRAP), metal ion chelating capacity (MH), and total antioxidant activity (TAA). The results listed in Table 5 show that LEO exhibits significant antioxidant activity, primarily due to its high content of oxygenated monoterpenes, especially linalool, linalyl acetate, and terpinen-4-ol. These compounds are extensively described in the literature for their free radical scavenging properties, their redox modulating effects, and their ability to interrupt oxidative chain reactions [58,59].

Lavender essential oil exhibited significant antioxidant properties in the CUPRAC test (42.53 mg TE/g), in the ABTS test (32.85 mg TE/g), in FRAP test (26.05 mg TE/g), and in DPPH test (1.28 mg TE/g), while the encapsulated formulation of lavender essential oil showed lower antioxidant potential of free essential oil. Interestingly, the chelating capacity of the metal ions of the microencapsulated oil was preserved (20.17 mg EDTA/g). This discrepancy in response between the assays reflects the multifactorial antioxidant mechanisms exerted by LEO, which include single electron transfer, hydrogen atom transfer, and metal ion complexation. The comparatively low activity measured in the DPPH assay is likely a consequence of steric hindrances due to the bulky structure of the terpene constituents or slower reaction kinetics in lipophilic, non-polar environments, which is characteristic of essential oils with many hydrophobic components [60].

The weakening of antioxidant activity after encapsulation can be attributed to several interrelated factors. During the encapsulation process, the hydroxyl group in the main components, including linalool, linalyl acetate, and terpinene-4-ol, can engage in hydrogen bonding with the hydroxyl group in maltodextrin. Consequently, the ability of encapsulated samples to donate electrons and hydrogen may be reduced compared to free essential oil [61,62].

As another insight, the physical entrapment of bioactive components in the encapsulation matrix reduces their molecular mobility and, consequently, their ability to directly scavenge reactive species during in vitro evaluation. In addition, spray drying exposes the oil to elevated temperatures and atmospheric oxygen, conditions that can promote thermal and oxidative degradation of thermolabile volatiles, particularly linalool and 1,8-cineole. Apart from these physical and thermal effects, encapsulation may cause molecular interactions between the essential oil components and the wall materials, such as maltodextrin and gum arabic, that alter the conformational stability, polarity, or redox potential of the active compounds. In some instances, these interactions can also favor secondary oxidative or hydrolytic reactions during storage, further reducing antioxidant efficacy over time [25,63].

Interestingly, although the overall antioxidant potential of encapsulated LEO3 was lower than that of the free oil, certain reactions (e.g., metal chelation capacity) were anticipated or even slightly enhanced. This can be explained by the stabilization of the sensitive terpenoid components against volatilization and oxidation, ensuring that their functional groups remain available for chelation. In addition, the controlled release of active compounds from the microcapsule matrix enables a more sustained interaction with reactive species, while carrier materials (maltodextrin and gum arabic) can improve the dispersion of hydrophobic molecules in aqueous systems and thus increase their effective activity. These mechanisms show that encapsulation can sometimes protect or even enhance specific antioxidant functions, although the overall capacity to scavenge radicals tends to decrease. Despite a measurable reduction in total antioxidant capacity, the technological and functional benefits of microencapsulation remain significant. By creating a robust physical barrier, the encapsulation matrix effectively shields sensitive volatiles from harmful environmental factors such as molecular oxygen, photodegradation, and thermal stress. This protective function significantly increases the chemical stability and shelf life of labile compounds during storage, transport, and industrial processing. In addition, microencapsulation offers the possibility of spatially targeted and temporally controlled release of bioactive components, whether in specific regions of the gastrointestinal tract, in the skin layers, or at other intended sites of action, thereby maintaining or even enhancing their functional efficacy at the site of administration [64]. It is also important to note that in vitro antioxidant assays may underestimate the preserved functional potential of encapsulated systems, as these methods do not fully account for the protective effects and targeted release mechanisms that manifest under physiological or application-specific conditions. Consequently, the real-life performance of encapsulated formulations, not only their in vitro activity, should be considered as the key parameter to assess their functional value.

To the best of our knowledge, there are no previously published studies specifically evaluating the antioxidant potential of microencapsulated essential oil of *Lavandula* × *intermedia*, which emphasizes the novelty and significance of the present results. In the absence of directly comparable data, the results obtained in this study were evaluated in the context of analogous studies on microencapsulation of other essential oils. A consistent trend was observed in the literature: the antioxidant activity of essential oils generally decreases after encapsulation compared to their unencapsulated counterparts. The trend observed in the present study is therefore consistent with existing findings and at the same time provides new insights into the specific behavior of *L.* × *intermedia* essential oil in encapsulated systems [65,66,67].

### 2.7. Enzyme Inhibition Potential

To investigate the potential functional properties of the free and microencapsulated essential oil of lavandin, the inhibitory activity against selected enzymes was analyzed. These tests included enzymes related to neurological functions (acetylcholinesterase, butyrylcholinesterase), melanin synthesis (tyrosinase), and carbohydrate metabolism (α-amylase and α-glucosidase). The results obtained are presented in Table 6 and show significant differences between the free and encapsulated forms of the oil, suggesting that the microencapsulation process can influence the bioactivity profile depending on the targeted enzyme and the type of active ingredients.

The strongest tyrosinase inhibitory activity of microencapsulated LEO3 was observed, with an inhibition value of 60.87 mg KAE/g, exceeding the activity of the free LEO (47.87 mg KAE/g). This result highlights a potential advantage of the encapsulation process in enhancing the bioavailability or controlled release of specific active constituents, such as linalool and linalyl acetate, which are known for their antityrosinase effects [68]. The observed increase in bioactivity can be attributed to several encapsulation-related mechanisms. Primarily, the encapsulation matrix serves to protect labile bioactive components from volatilization and degradation, thereby increasing their chemical stability and efficiency in inhibiting excessive enzyme activity. In addition, the controlled release profile of the microcapsules facilitates prolonged and sustained interaction with the target enzymes, thereby enhancing the inhibitory effect over longer periods. There is also evidence in the literature that certain wall materials, particularly those enriched with proteins or polysaccharides, can interact synergistically with phenolic and terpene compounds, further enhancing their functional performance [26].

While a reduction in some activities was observed after encapsulation, other activities, such as the inhibition of tyrosinase, were enhanced. This apparent improvement could be due to the fact that the main bioactive ingredients are better preserved in the encapsulation matrix and are therefore not degraded under the test conditions. In addition, the microcapsule structure provides a controlled release profile that allows a longer and more effective interaction with the active sites of the enzymes. The interactions between the wall materials and the terpenoid compounds could also stabilize the bioactive conformations or modify the solubility and thus improve the accessibility for the enzymes. Taken together, these effects explain why encapsulated LEO may in some cases have a higher inhibitory potential than the free essential oil [28]. In contrast, inhibition of acetylcholinesterase (AChE) and butyrylcholinesterase (BChE) was comparable or slightly higher in the free LEO. For AChE, only the free oil showed measurable activity (2.69 mg GALAE/g), while BChE inhibition was nearly equivalent in both forms (2.77 mg GALAE/g in free LEO and 2.92 mg GALAE/g in encapsulated LEO). The lack of AChE inhibition in the microencapsulated sample suggests that encapsulation may limit the immediate bioavailability of compounds responsible for AChE inhibition or that such activity is sensitive to processing conditions.

As for carbohydrate-hydrolyzing enzymes, free LEO showed a moderate inhibition of α-amylase (0.42 mmol ACAE/g), while this activity was significantly reduced in the encapsulated sample (0.13 mmol ACAE/g). The decrease can be explained by the physical entrapment of the active components in the carrier matrix, limiting their direct access to the enzyme binding sites. Similar results were reported for other encapsulated essential oils, where the inhibition of α-amylase decreased after encapsulation due to reduced molecular availability. In contrast, the inhibition of α-glucosidase was maintained after encapsulation, with free and encapsulated LEO showing the same activity (1.64 mmol ACAE/g). This retention of activity suggests that the compounds responsible for α-glucosidase inhibition remain stable and bioavailable within the microcapsules. As α-glucosidase inhibition plays a key role in moderating postprandial blood glucose levels, the retention of this property emphasizes the potential of lavandin oil microcapsules for functional food or nutraceutical applications aimed at controlling blood glucose levels [28]. As there are few studies specifically investigating the enzyme inhibitory potential of lavender essential oil microcapsules, the bioactivity of the free essential oil was contextualized by comparison with existing literature data on related species. In particular, *Lavandula officinalis* was shown to effectively inhibit AChE enzyme, with reported IC_50_ values ranging from 0.18 to 0.46 mg/mL [69]. In addition, in the study [70], *Lavandula pinnata* showed an inhibitory effect on several enzymes involved in metabolic regulation, including tyrosinase (IC_50_ = 29.11 mg/mL), α-amylase (IC_50_ = 31.56 µg/mL), and α-glucosidase (IC_50_ of 58.47 µg/mL). The findings presented here demonstrate that lavandin essential oil exerts an inhibitory effect on several enzyme targets, with microencapsulation profoundly modulating its biological activity profile. Beyond the encapsulation process, the extraction technique used exerts a decisive influence on the chemical composition of the essential oil, with the presence and relative abundance of secondary metabolites, particularly terpene constituents, critically determining the bioactivity of both the free and encapsulated forms.

## 3. Materials and Methods

### 3.1. Material

Lavender (*Lavandula* × *intermedia* ‘Grosso’) was cultivated in the agricultural cooperative “Lavanda” in village Sviloš, Fruška Gora mountain, North Serbia. The homemade essential oils were produced locally in the agricultural cooperative by steam distillation. Gum arabic (GA1 and GA2) from acacia trees and low viscosity maltodextrin (DE 16–20, MD) were purchased from Sigma Aldrich (Steinheim, Germany). Two types of GA were used: GA1 (additionally processed powder by spray drying) and GA2 (branched polysaccharide powder). Whey protein concentrate (WPC) was purchased from Lactoprot (Kaltenkirchen, Germany), inulin (IN) from chicory was purchased from Fornatura d.o.o. (Belgrade, Serbia) and modified starch from waxy maize (Hi-Cup 100) was purchased from National Starch and Chemical (Manchester, UK). Ethanol (Merck, Darmstadt, Germany), hexane (CARLO ERBA Reagents, Sabadell, Spain) and petroleum ether (VWR International S.A.S., Fontenay-sous-Bois, Paris, France) used for the experiments were of analytical grade.

### 3.2. GC/MS Analysis

The sample preparation of LEOs was carried out by diluting a certain amount of crude oil with n-hexane (high purity). The dilution factor was 10,000 and the essential oils were analyzed using an Agilent 7890 B gas chromatograph coupled to an Agilent 5977 mass spectrometer. For the chromatographic separation, an HP-5 capillary column (length 30 m, 0.25 mm inner diameter, 0.25 µm film thickness Agilent Technologies, Santa Clara, CA, USA) was used with He (99.999%) as a carrier gas at a constant flow rate of 1 mL/min. The injection volume of the diluted sample was 2 µL using the splitless mode of sample injection. The injector and transfer line temperatures were maintained at 220 and 270 °C, respectively. The initial oven temperature was 60 °C and was then gradually increased to 246 °C at 3 °C/min. The ion source temperature was maintained at 230 °C, the electron energy at 70 eV, and the quadrupole temperature at 150 °C. Full spectral information (SCAN mode) was collected for the *m*/*z* range of 35–500 u. The compounds were identified by comparing the mass spectra with libraries (US National Institute of Standards and Technology, NIST, and Mass Hunter Workstation Software, Version B.06.00 SP01/Build 6.0.388.1, Unknown Analysis), and confirmed by comparison of calculated Kovats retention indices (linear temperature-programmed retention index (LTPRI)) with literature data [71]. For retention indices estimation, the homologous series of the n-alkane mixture consisting of a mixture of C_9_–C_28_ n-alkanes corresponding to 820–2800 KI was used. Relative amounts of components, expressed in percentages (%, *m*/*m*), were calculated by normalization measurement according to the peak area in the total ion chromatogram.

### 3.3. Preparation of Oil-in-Water Emulsions

The solutions of the wall materials in demineralized water were prepared the day before and kept overnight at 4 °C to ensure complete hydration of the molecules. The core material consisted of LEO and was kept at a constant level of 5% in all experiments. The experiments were performed with different wall material formulations of LEO emulsions (Table 7). During emulsification, LEO was added dropwise into the solution of the wall material and mixed with a homogenizer (Ultra-turrax, IKA, Staufen, Germany) at 10,000 rpm for 5 min. The ratio of core to wall material was 1:4 (*w*/*w*) in all formulations. The freshly prepared emulsions were used as feed solutions for the spray-drying process.

### 3.4. Preparation of Microcapsules

The microcapsules were prepared by injecting the feed emulsion into a laboratory spray dryer (Büchi 190 Mini Spray Dryer, Büchi, Uster, Switzerland) equipped with a nozzle with a diameter of 0.7 mm under the following conditions: air inlet temperature 140 °C, air outlet temperature 95 °C and flow rate of a peristaltic pump 3.2 mL/min. The prepared microcapsules were collected and stored in desiccators at room temperature until their characterization.

### 3.5. Characterization of LEO Microcapsules

The encapsulation yield was determined by measuring the mass of the collected powder after the spray drying process (W_MK_) and calculated as follows (Equation (1)):

(1) $$Yield=\frac{W_{MK}}{W_{1}}\times 100\;\;\;[\%]$$

where W_1_ was the mass of the total solids content in the feed emulsion [27].

The surface LEO content in microcapsules was performed according to the described method: five grams of microcapsules were gently stirred with 10 mL of n-hexane for 10 min, then the suspension was filtered and washed three times with 5 mL of petroleum ether. The powder was dried in an oven at 90 °C to a constant weight. The surface oil content (W_SO_) was expressed as the difference between the initial weight of the microcapsules and the mass of the dried powder [33].

The total oil content of LEO loaded in the microcapsules is the sum of the oil in the surface of the microcapsules and the oil trapped in the wall material of the microcapsule. The total oil was determined by distillation in a Clevenger apparatus for 3 h, mixing 5 g of the powder with 250 mL of demineralised water. The total oil weight (W_TO_) in the microcapsules was determined by multiplying the volume of oil contained in the measuring burette by the oil density (0.8976 g/cm^3^ at 20 °C) [33].

The oil retention efficiency (RE) was defined as the percentage of oil loaded into the microcapsules after spray drying concerning the original oil mass added to the feed emulsion (W_IO_) (Equation (2)), and the encapsulation efficiency (EE) of LEO was defined as the ratio between the oil encapsulated in a wall material and the total oil loaded into the microcapsules (Equation (3)):

(2) $$RE=\frac{W_{TO}}{W_{IO}}\times 100\;\;\;[\%]$$

(3) $$EE=\frac{W_{TO}-W_{SO}}{W_{TO}}\times 100\;\;\;[\%]$$

where W_TO_ is the total oil mass obtained by distillation and W_SO_ is the surface oil mass obtained by extraction.

The moisture content of the LEO powder was determined gravimetrically according to the AOAC method (AOAC 2005) [31]. One gram of the powder was placed in a glass container and dried at 105 °C to constant weight.

The hygroscopicity of the LEO powder was determined according to paper [72]. One gram of the powder was stored in desiccators with a saturated sodium chloride solution (75.29% relative humidity at 25 °C) for 7 days. The hygroscopicity of the microcapsules was calculated as the percentage ratio of the mass of adsorbed moisture to the mass of the measured microcapsules.

The dissolution time of the microcapsules was measured according to the method proposed by study [36]. For this purpose, 0.5 g of the microcapsules was added to 10 mL of water, and the time until complete dissolution was measured while stirring at 700 rpm with a magnetic stirrer.

The bulk density (ρ_B_) of the powder was determined according to paper [50]. Approximately 2 g of the microcapsule powder was poured into a 10 mL measuring cylinder and the mass and volume of the microcapsules were then measured. The glass cylinder containing the powder was tapped on a solid surface for approximately 3 min until a constant volume of powder was present in the cylinder. The tapped density (ρ_T_) was the ratio between the weight of the powder and the volume of the compressed powder [33].

The particle density (ρ_P_) of the LEO microcapsules was determined according to paper [50]. One gramme of microcapsules was poured into a 10 mL glass cylinder with a lid and shaken for 30 s with 5 cm^3^ petroleum ether. The microcapsules remaining on the cylinder wall were washed with 2 cm^3^ petroleum ether. The particle density was calculated using Equation (4):

(4) $$\rho_{P}=\frac{W_{M}}{V_{total}-V_{PE}}\;\;\;[g/{cm}^{3}]$$

where W_M_ is the mass of the microcapsule powder, V_total_ is the measured volume of the solvent and powder, V_PE_ is the volume of the petroleum ether [33].

The flowability and cohesiveness of the LEO microcapsules were estimated based on Carr’s index (CI) and Hausner ratio (HR) according to Equations (Equations (5) and (6)) [36]:

(5) $$CI=\frac{\left(\rho_{T}-\rho_{B}\right)}{\rho_{T}}\times 100$$

(6) $$HR=\frac{\rho_{T}}{\rho_{B}}$$

Porosity of the powder was calculated according to Equation (7):

(7) $$Porosity=\frac{\rho_{P}-\rho_{T}}{\rho_{P}}\times 100\;\;\;[\%]$$

The particle size and microstructural properties of microcapsules were analyzed using laser light diffraction with a Mastersizer 2000 (Malvern Instruments, Malvern, UK), operating in dry mode, and the field emission scanning electron microscope (SEM, ThermoFisher Scientific Apreo 2C, Waltham, MA, USA).

### 3.6. Microcapsules Thermal Analysis

Thermal analysis of free LEO, wall materials and LEO microcapsules was performed on the Netzsch STA 449 F5 Jupiter instrument (NETZSCH-Gerätebau GmbH, Selb, Germany). Approximately 15 mg of the sample was analyzed by heating from room temperature to 600 °C at a heating rate of 10 °C/min.

### 3.7. Microcapsule Antimicrobial Activity

The antimicrobial activity of free LEO and microencapsulated LEO was tested on selected bacteria and fungi. The most common representatives of Gram-positive bacteria: *Bacillus cereus*, *Enterococcus faecalis* and *Staphylococcus aureus*, as well as pathogenic representatives of Gram-negative bacteria: *Salmonella enterica*, *Escherichia coli* and *Klebsiella pneumoniae*, were selected. The activity of free LEO and microencapsulated LEO on the listed bacteria was tested by the disk-diffusion method in Petri dishes with a diameter of 90 mm. The method is described in detail in the paper [73]. Briefly, the appropriate test bacteria on the Nutrient agar medium were previously seeded in Petri dishes. After the medium was solidified, sterile disks with a diameter of 5 mm (HiMedia, Mumbai, India) were applied to the surface. The test sample containing LEO was applied to sterile disks with a micropipette in an amount of 5 µL. After incubation for 48 h at 30 °C, the inhibition zones were measured with a special ruler. All experiments were performed in three replicates.

On the other hand, significant representatives of fungal pathogens were also selected for testing. Selected fungi are producers of mycotoxins that, according to the International Agency for Research on Cancer, belong to the group of highly toxic to humans [74]: *Aspergillus flavus*, *Fusarium graminearum* and *Penicillium* sp. The effects of free LEO and microencapsulated LEO on these fungi were tested using the well-diffusion method described in detail in the paper [75]. Briefly, for each treatment, 100 µL of the LEO sample was added to each well. As a negative control treatment, sterile distilled water was used. The test was conducted in three repetitions. Antagonistic activity was determined after 5 days of incubation at 27 °C by measuring zones formed around wells with no visible fungal mycelia growth.

Free LEO was used in its native form for testing, while microcapsules were dissolved in sterile distilled water before use to final concentrations of 1–5% (*w*/*v*) relative to the oil content in the microcapsules. The results were measured after a period of incubation of Petri dishes under defined conditions. Each experiment was performed in triplicate.

### 3.8. Antioxidant and Enzyme Inhibitor Activity

In the applied tests, the free LEO was first dissolved in ethanol to obtain a stock solution with a concentration of 2 mg/mL, which was then used directly in the tests. For the encapsulated sample (LEO3), the amount of powder required was calculated based on the encapsulation efficiency (78.77%) to ensure that the final concentration of lavandin essential oil in ethanol was equal to that of the free oil (2 mg/mL). Ethanol was chosen as the solvent due to its compatibility with enzyme inhibition and antioxidant assays, its solubility of hydrophobic essential oil components and the reproducibility of the measurements. The antioxidant capacity of LEO and LEO3 was evaluated using DPPH, ABTS, CUPRAC, FRAP, MC and TTA assays, with results expressed as mg Trolox equivalents (TE)/g oil for DPPH, ABTS, CUPRAC and FRAP, mg EDTA equivalents (EDTAE)/g for MC and mmol TE/g for TTA. The enzyme inhibitory activities were tested against acetylcholinesterase, butyrylcholinesterase, tyrosinase, α-amylase and α-glucosidase using galanthamine, kojic acid and acarbose as reference standards, and the results were expressed as GALAE, KAE and ACAE equivalents per g of essential oil, respectively [76,77]. All experiments were performed in triplicate and results are expressed as effective essential oil concentration.

### 3.9. Statistical Analysis

The data were analyzed using analysis of variance (ANOVA) and significant differences (*p* ≤ 0.05) between the mean values were determined with the TUKEY HSD test using Statistica 14.0 software (TIBCO Software Inc., Palo Alto, CA, USA). All experiments were performed in triplicate and the results were averaged.

## 4. Conclusions

This study has shown that spray drying is an effective strategy to stabilize and functionally improve the essential oil of lavandin (*Lavandula* × *intermedia*). The selection of wall material significantly impacted encapsulation performance, with encapsulation efficiencies spanning from 55.35% to 83.29%, and oil retention ranging between 49.07% and 76.65%. Among the formulations assessed, the system comprising maltodextrin/gum arabic with Tween 80 (LEO3) emerged as the most balanced, demonstrating high encapsulation efficiency, desirable powder characteristics, robust thermal stability, and enhanced moisture resistance. GC–MS analysis verified that linalyl acetate and linalool were the primary components among the active ingredients. Biological evaluations indicated that while antioxidant activity decreased post-encapsulation, antimicrobial and antifungal activities remained consistent, and tyrosinase inhibition was improved in the microencapsulated samples. These findings underscore that microencapsulation not only preserves the volatile and sensitive components of LEO but also alters its bioactivity, facilitating its incorporation into pharmaceutical, food, and cosmetic fields. Future research should aim to optimize wall material combinations, scale up production, and assess in vivo effectiveness to fully leverage the industrial potential of microencapsulated lavandin essential oil.

## Figures and Tables

**Table 2:** Total oil content, surface oil content, oil retention efficiency (RE), encapsulation efficiency (EE) and powder yield (Y) of the LEO microcapsules.

Parameters	Total Oil Content (g 100 g^−1^)	Surface Oil Content (g 100 g^−1^)	Oil Retention (%)	Encapsulation Efficiency (%)	Yield (%)
LEO1 (MD)	13.54 ± 0.18 ^b,c^	2.28 ± 0.01 ^f^	67.71 ± 0.89 ^b,c^	83.29 ± 0.10 ^a^	71.47 ± 1.03 ^a^
LEO2 (MD/GA2)	13.26 ± 0.54 ^c^	4.63 ± 0.01 ^b^	66.29 ± 2.72 ^c^	65.03 ± 1.37 ^e^	44.50 ± 1.14 ^d,e^
LEO3 (MD/GA1)	14.28 ± 0.07 ^b^	3.02 ± 0.10 ^d^	71.40 ± 0.36 ^b^	78.77 ± 0.72 ^b^	41.39 ± 2.18 ^e^
LEO4 (MD/GA1)	13.66 ± 0.06 ^b,c^	4.15 ± 0.01 ^c^	68.28 ± 0.28 ^b,c^	69.55 ± 0.08 ^d^	47.53 ± 0.69 ^d^
LEO5 (MD/WPC)	12.08 ± 0.14 ^d^	2.73 ± 0.11 ^e^	60.41 ± 0.72 ^d^	77.37 ± 1.2 ^b^	55.56 ± 0.36 ^c^
LEO6 (MD/IN)	9.81 ± 0.11 ^e^	2.69 ± 0.02 ^e^	49.07 ± 0.55 ^e^	72.34 ± 0.18 ^c^	65.51 ± 1.31 ^b^
LEO7 (GA1/IN)	13.31 ± 0.17 ^c^	5.94 ± 0.06 ^a^	66.56 ± 0.62 ^b,c^	55.35 ± 0.41 ^f^	53.11 ± 0.62 ^c^
LEO8 (Hi-Cup)	15.33 ± 0.15 ^a^	4.52 ± 0.05 ^b^	76.65 ± 0.25 ^a^	70.54 ± 0.35 ^c,d^	55.54 ± 1.22 ^c^

^a,b,c,d,e,f^ Values with different letters in the same column differ significantly (*p* ≤ 0.05).

**Table 3:** Moisture, dissolution time, bulk density, tapped density, particle density, Carr’s index (CI), Hausner ratio (HR) and porosity of *Lavandula* × *intermedia* essential oil (LEO) microcapsules.

Parameters	Moisture (%)	Dissolution Time (min)	Bulk Density (g/cm^3^)	Tapped Density (g/cm^3^)	Particle Density (g/cm^3^)	CI (%)	HR	Porosity (%)
LEO1 (MD)	1.21 ± 0.15 ^a^	0.82 ± 0.13 ^a^	0.269 ± 0.018 ^a,b^	0.534 ± 0.018 ^a^	0.85 ± 0.04 ^a^	51.81 ± 1.77 ^e^	2.08 ± 0.08 ^d^	37.68 ± 4.98 ^a^
LEO2 (MD/GA2)	4.33 ± 0.12 ^c^	3.99 ± 0.20 ^b^	0.240 ± 0.001 ^b^	0.440 ± 0.001 ^c^	1.44 ± 0.01 ^c^	45.26 ± 0.04 ^b,c,d^	1.83 ± 0.01 ^b,c^	69.46 ± 0.14 ^c,d^
LEO3 (MD/GA1)	2.28 ± 0.01 ^c^	3.42 ± 0.58 ^b^	0.251 ± 0.004 ^a,b^	0.450 ± 0.003 ^b,c^	1.23 ± 0.08 ^b^	44.15 ± 0.05 ^b,c^	1.79 ± 0.00 ^b,c^	63.48 ± 2.44 ^b,c^
LEO4 (MD/GA1)	4.63 ± 0.07 ^d,e^	3.64 ± 0.25 ^b^	0.244 ± 0.002 ^b^	0.480 ± 0.003 ^b^	1.83 ± 0.01 ^e^	50.56 ± 0.78 ^e^	2.02 ± 0.03 ^c,d^	73.92 ± 0.18 ^d^
LEO5 (MD/WPC)	2.25 ± 0.14 ^c^	6.93 ± 0.27 ^c^	0.230 ± 0.001 ^b^	0.404 ± 0.003 ^d^	0.97 ± 0.01 ^a^	43.15 ± 0.04 ^b^	1.76 ± 0.00 ^b^	59.31 ± 1.20 ^b^
LEO6 (MD/IN)	1.12 ± 0.06 ^a^	0.98 ± 0.09 ^a^	0.281 ± 0.001 ^a^	0.462 ± 0.009 ^b,c^	1.43 ± 0.01 ^c^	38.86 ± 0.03 ^a^	1.63 ± 0.014 ^a^	67.92 ± 0.15 ^c,d^
LEO7 (GA1/IN)	4.92 ± 0.27 ^e^	3.87 ± 0.17 ^b^	0.240 ± 0.001 ^b^	0.450 ± 0.003 ^b,c^	1.67 ± 0.02 ^d^	46.63 ± 0.03 ^c,d^	1.88 ± 0.01 ^b,c^	72.81 ± 0.34 ^d^
LEO8 (Hi-Cup)	1.59 ± 0.03 ^b^	3.31 ± 0.04 ^b^	0.253 ± 0.001 ^b^	0.473 ± 0.002 ^b,c^	1.45 ± 0.01 ^c^	47.34 ± 0.07 ^d^	1.89 ± 0.00 ^c^	67.93 ± 0.10 ^c,d^

^a,b,c,d,e^ Values with different letters in the same column differ significantly (*p* ≤ 0.05).

**Table 4:** Antimicrobial and antifungal activity (mm) of free lavandin essential oil (LEO, tested as 100% oil) and microencapsulated LEO reconstituted to 1–5% (*w*/*v*) oil content.

LEO3 Concentration (%)	1 *	2 *	3 *	4 *	5 *	LEO
*Bacillus cereus*	n.a.	n.a.	12.00 ± 0.50 ^b^	12.50 ± 0.71 ^c^	13.00 ± 0.50	21.75 ± 2.36 ^c^
*Enterococcus faecalis*	12.50 ± 0.71	13.00 ± 0.50 ^a^	14.50 ± 0.71 ^b^	15.00 ± 0.50 ^b^	15.00 ± 0.50 ^b^	14.25 ± 0.96 ^d^
*Salmonella enterica*	n.a.	n.a.	n.a.	10.50 ± 0.71 ^c^	11.50 ± 0.71 ^c^	21.50 ± 2.12 ^c^
*Staphiloccus aureus*	n.a.	n.a.	n.a.	11.50 ± 0.07 ^c^	12.20 ± 0.29 ^c^	24.50 ± 1.92 ^c^
*Escherichia coli*	n.a.	n.a.	n.a.	n.a.	11.50 ± 0.71 ^c^	43.50 ± 1.00 ^a^
*Klebsiella pneumoniae*	n.a.	10.50 ± 0.71 ^b^	14.00 ± 0.50 ^b^	14.5 ± 0.71 ^b^	16.00 ± 1.41 ^b^	20.75 ± 0.96 ^c^
*F. graminearum*	n.a.	n.a.	n.a.	n.a.	n.a.	40.75 ± 2.63 ^a^
*A. flavus*	n.a.	n.a.	n.a.	20.50 ± 0.71 ^a^	21.50 ± 0.71 ^a^	21.50 ± 0.58 ^c^
*Penicillium* sp.	n.a.	n.a.	20.50 ± 0.71 ^a^	21.00 ± 0.50 ^a^	22.00 ± 0.50 ^a^	38.75 ± 2.87 ^a,b^

Values are means ± SD of three measurements, means within each row with different letters (^a–d^) differ significantly (*p* ≤ 0.05); * Concentration of LEO in an emulsion prepared from microcapsules; n.a.—not active

**Table 5:** Antioxidative activity of free LEO and encapsulated LEO3.

		LEO	LEO3
Antioxidant assays	DPPH (mg TE/g)	1.28 ± 0.25	n.a.
	ABTS (mg TE/g)	32.85 ± 2.01	n.a.
	CUPRAC (mg TE/g)	42.53 ± 1.05 ^a^	12.00 ± 0.97 ^b^
	FRAP (mg TE/g)	26.05 ± 2.11 ^a^	9.81 ± 0.54 ^b^
	MC (mg EDTA/g)	n.a.	20.17 ± 0.48
	TAA (mmolTE/g)	72.43 ± 0.04 ^a^	0.99 ± 0.04 ^b^

Values are means ± SD of three measurements, means within each row with different letters (^a,b^) differ significantly (*p* ≤ 0.05); n.a.—not active.

**Table 6:** Enzyme inhibition potential of free LEO and encapsulated LEO3.

**Enzyme** **inhibitor** **assays**		**LEO**	**LEO3**
	AChE (mg GALAE/g)	2.69 ± 0.14	n.a.
	BChE (mg GALAE/g)	2.77 ± 0.11 ^a^	2.92 ± 0.19 ^a^
	Tyrosinase (mg KAE/g)	47.87 ± 6.54 ^b^	60.87 ± 2.02 ^a^
	α-Amylase (mmol ACAE/g)	0.42 ± 0.05 ^a^	0.13 ± 0.001 ^b^
	α-Glucosidase (mmol ACAE/g)	1.64 ± 0.004 ^a^	1.64 ± 0.006 ^a^

Values are means ± SD of three measurements, means within each row with different letters (^a,b^) differ significantly (*p* ≤ 0.05); n.a.—not active.

**Table 7:** Composition of wall materials for microencapsulation of *Lavandula* × *intermedia* essential oil by spray drying.

	Wall Material (g 100 g^−1^)						Tween 80 (% (*w*/*v*))	Core Material (g 100 g^−1^)
	MD	GA1	GA2	WPC	IN	Hi-Cup		
LEO1	20	-	-	-	-	-	2	5
LEO2	10	-	10	-	-	-	-	5
LEO3	10	10	-	-	-	-	2	5
LEO4	10	10	-	-	-	-	-	5
LEO5	10	-	-	10	-	-	-	5
LEO6	10	-	-	-	10	-	2	5
LEO7	-	10	-	-	10	-	-	5
LEO8	-	-	-	-	-	20	-	5

## Data Availability

The original contributions presented in the study are included in the article. Further inquiries can be directed to the corresponding author.

## Abbreviations

The following abbreviations are used in this manuscript:

EOs	Essential oils
CAGR	Compound annual growth rate
LEO	Lavandin essential oil
RE	Retention efficiency
EE	Encapsulation efficiency
GC-MS	Gas chromatography–mass spectrometry
Y	Yield
MD	Maltodextrin
GA	Gum arabica
WPC	Whey protein concentrate
IN	Inulin
Hi-Cup	Modified starch
CI	Carr’s index
HR	Hausner ratio
TG	Thermogravimetric analysis
DDPH	2,2-diphenyl-1-picryl-hydrazyl-hydrate
ABTS	2,2′-azino-bis (3-ethylbenzothiazoline-6-sulphonic acid)
CUPRAC	Cupric-reducing antioxidant capacity
FRAP	Ferric-reducing antioxidant power
MC	Metal chelating assay
TAA	Total antioxidant activity
TE	Trolox equivalents
EDTA	Ethylenediaminetetraacetic acid equivalents
AChE	Acetylcholinesterase
BChE	Butyrylcholinesterase
GALAE	Galantamine equivalents
KAE	Kojic acid equivalents
ACAE	Acarbose equivalent
